# Phase-controllable growth of ultrathin 2D magnetic FeTe crystals

**DOI:** 10.1038/s41467-020-17253-x

**Published:** 2020-07-24

**Authors:** Lixing Kang, Chen Ye, Xiaoxu Zhao, Xieyu Zhou, Junxiong Hu, Qiao Li, Dan Liu, Chandreyee Manas Das, Jiefu Yang, Dianyi Hu, Jieqiong Chen, Xun Cao, Yong Zhang, Manzhang Xu, Jun Di, Dan Tian, Pin Song, Govindan Kutty, Qingsheng Zeng, Qundong Fu, Ya Deng, Jiadong Zhou, Ariando Ariando, Feng Miao, Guo Hong, Yizhong Huang, Stephen J. Pennycook, Ken-Tye Yong, Wei Ji, Xiao Renshaw Wang, Zheng Liu

**Affiliations:** 1 School of Materials Science and Engineering, Nanyang Technological University, Singapore, 639798 Singapore; 2 CINTRA CNRS/NTU/THALES, UMI 3288, Research Techno Plaza, Singapore, 637553 Singapore; 3 School of Physical and Mathematical Sciences, Nanyang Technological University, Singapore, 639798 Singapore; 4 Department of Materials Science and Engineering, National University of Singapore, Singapore, 117575 Singapore; 5 Department of Physics and Beijing Key Laboratory of Optoelectronic Functional Materials & Micro-Nano Devices, Renmin University of China, 100872 Beijing, China; 6 Department of Physics, National University of Singapore, Singapore, 117551 Singapore; 7 National Laboratory of Solid State Microstructures, School of Physics, Collaborative Innovation Center of Advanced Microstructures, Nanjing University, Nanjing, 210093 China; 8 Institute of Applied Physics and Materials Engineering, University of Macau, Macau, SAR 999078 China; 9 Department of Physics and Chemistry, Faculty of Science and Technology, University of Macau, Macau, SAR 999078 China; 10 Centre for Micro-/Nano-electronics (NOVITAS), School of Electrical and Electronic Engineering, Nanyang Technological University, Singapore, 639798 Singapore

**Keywords:** Materials science, Materials science, Nanoscience and technology, Nanoscience and technology, Physics

## Abstract

Two-dimensional (2D) magnets with intrinsic ferromagnetic/antiferromagnetic (FM/AFM) ordering are highly desirable for future spintronic devices. However, the direct growth of their crystals is in its infancy. Here we report a chemical vapor deposition approach to controllably grow layered tetragonal and non-layered hexagonal FeTe nanoplates with their thicknesses down to 3.6 and 2.8 nm, respectively. Moreover, transport measurements reveal these obtained FeTe nanoflakes show a thickness-dependent magnetic transition. Antiferromagnetic tetragonal FeTe with the Néel temperature (*T*_N_) gradually decreases from 70 to 45 K as the thickness declines from 32 to 5 nm. And ferromagnetic hexagonal FeTe is accompanied by a drop of the Curie temperature (*T*_C_) from 220 K (30 nm) to 170 K (4 nm). Theoretical calculations indicate that the ferromagnetic order in hexagonal FeTe is originated from its concomitant lattice distortion and Stoner instability. This study highlights its potential applications in future spintronic devices.

## Abstract (web summary)

Two-dimensional magnets with intrinsic ferromagnetic/antiferromagnetic ordering are highly desirable for future spintronic devices. Here, the authors demonstrate a chemical vapor deposition approach to controllably grow ultrathin FeTe crystals with antiferromagnetic tetragonal and ferromagnetic hexagonal phase, showing a thickness-dependent magnetic transition.

## Introduction

Recently, 2D magnets have drawn much attention because they provide an ideal platform for exploring magnetism down to atomic-layer thicknesses^1,2^. The discovery of long-range magnetic order in 2D van der Waals (vdW) crystals is vital to the understanding of the spin behavior in 2D limit and can enable extensive spintronic applications ranging from molecular quantum devices to high-density data storage devices^3–5^. Different from conventional magnetic thin films and their bulk counterparts, these intrinsic 2D magnetic crystals show very peculiar properties and often bring about different physical phenomena. For instance, layer dependent ferromagnetism with an out-of-plane anisotropy was observed in CrI_3_ and Cr_2_Ge_2_Te_6_ nanosheets at low temperatures^3,6^. Furthermore, ferromagnetic ordering with Curie transition temperature (*T*_c_) > 300 K was induced in Fe_3_GeTe_2_ extrinsically by an ionic gating method^7^. In addition, long-distance magnon transport was also detected in the antiferromagnetic MnPS_3_ crystal^8^.

However, two issues hinder the practical applications of 2D magnets. First, some 2D magnets are extremely unstable (e.g., CrI_3_). Therefore, either glove box-involved mechanical exfoliation or molecular beam epitaxial (MBE) method has to be employed to prepare the 2D magnets in an oxygen/water-free or ultra-high vacuum condition. Also, these approaches are time-consuming and not industrial scalable^9,10^. Second, many 2D magnets show intricate phases (e.g., magnetic sulfides/selenides and alloy). Hence, it is challenging to identify an exact phase of these 2D magnets and provide a sound explanation for the origin of its magnetism. Recently, selective substrates, such as mica, were chosen for the growth of a few of 2D magnets, including ultrathin Cr_2_S_3_ and CrSe crystals^11,12^. Such epitaxial growth technique can only be applied to a limited number of 2D magnets and it also raises a transfer problem during the device fabrication or magnetic measurements. For instance, the transfer process inevitably introduces defects and contamination, which dramatically degrades the performance of devices. Thus, exploring more 2D magnetic compounds and developing reliable synthesis methods are urgent and necessary.

Fe-chalcogenides (FeS, FeSe, and FeTe) are a recent class of magnetic materials^13,14^. By changing the chalcogen elements (S, Se, and Te), a variety of magnetic behaviors, such as ferromagnetism, ferrimagnetism, and antiferromagnetism can be observed^15,16^. In addition, Fe-chalcogenides also exhibit multiple structural phases with distinct properties^17,18^. For instance, the conventional phase of FeTe is an antiferromagnetic metal with a tetragonal crystal structure and *T*_N_ ≈ 70 K^16^. Recently, density-functional theory (DFT) calculations predicted an intriguing hexagonal FeTe phase, which is weakly magnetic^19^. As the magnetic properties of FeTe can be significantly affected by the changes in structural phases, controlled synthesis of FeTe with tunable structural phases is needed. So far, the synthesis of 2D FeTe crystals by CVD method has been rarely reported, much less the phase control^20^.

In this work, we have directly synthesized antiferromagnetic and ferromagnetic FeTe on SiO_2_/Si substrates. By controlling the growth temperature, ultrathin 2D-layered tetragonal FeTe nanoplates and non-layered hexagonal FeTe nanoplates were selectively obtained as square or triangular shapes, with thicknesses down to 2.8 and 3.6 nm, respectively. The grain size of FeTe flakes is up to dozens of micrometers, suggesting they are high-quality single crystals. Furthermore, the antiferromagnetic behavior of tetragonal FeTe is exhibited with a *T*_N_ decreases as the thickness decline, while the ferromagnetic hexagonal FeTe is also displayed with a thickness-dependent *T*_C_. Theoretical calculations indicate that the structural distortion is responsible for the observed ferromagnetism.

## Results

### Phase-tunable growth of FeTe crystals

**Fig. 1:**
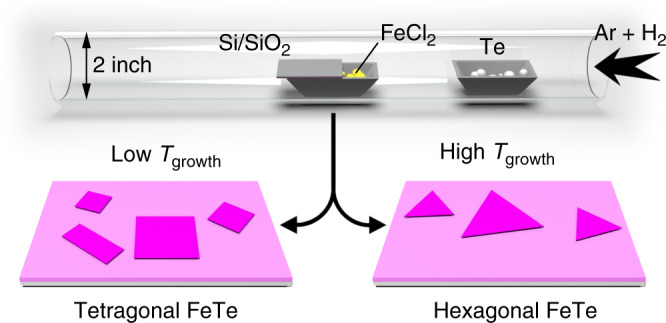
Illustration of the experimental setup. The schematic view for the iron tellurides growth process.

Figure 1 schematically illustrates the setup of the ambient pressure CVD system for the growth of FeTe crystals on SiO_2_/Si substrates. Briefly, ferrous chloride (FeCl_2_) and tellurium (Te) powders were used as reactants. During the CVD process, the Ar and H_2_ mixture carries a certain amount of tellurium vapor at a fixed heating temperature of tellurium source (*T*_Te_) to react with evaporated FeCl_2_ at different growth temperatures (*T*_growth_) to produce different phases. Details about the sample synthesis are provided in the Methods section. As shown in Supplementary Fig. 1, the tetragonal FeTe belongs to *P*4*/nmm* space group owning a layered structure in which Fe atom layer and the double slabs of Te atom layers are interlaced in the interlayer direction (Supplementary Fig. 1a). While hexagonal FeTe has a non-layered structure that belongs to *P*6_3_*/mmc* space group. This structure can be regarded as the closed-packed Fe atomic planes alternatively occupying the octahedral vacancies created by the *AB*-stacked Te atomic planes (Supplementary Fig. 1b).

**Fig. 2:**
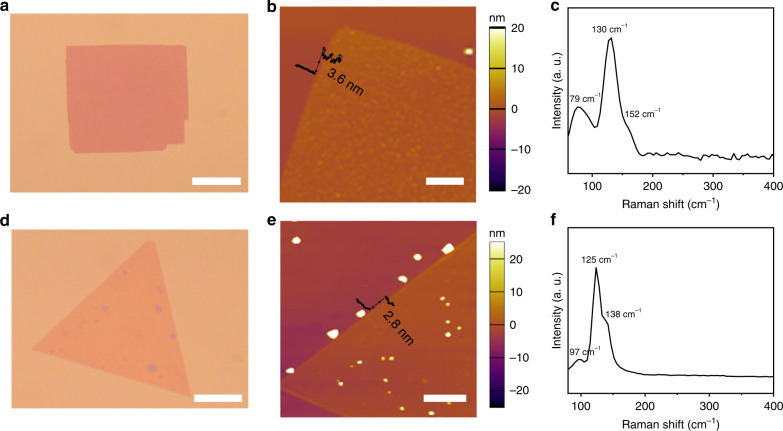
Morphological and structural characterization of the as-synthesized FeTe samples. **a**, **d** Typical optical images of as-grown tetragonal (**a**) and hexagonal (**d**) FeTe crystals on SiO_2_/Si substrates. **b**, **e** AFM images of the square FeTe nanoplate with a thickness of 3.6 nm and the trigonal FeTe nanoplate with a thickness of 2.8 nm. **c**, **f** Typical Raman spectra of tetragonal and hexagonal FeTe nanoplates. Scale bars: 20 μm in **a**, **d**; 2 μm in **b**; 3 μm in **e**.

One of the most important features of FeTe is its phase tunability  which originates from the formation energy difference between the hexagonal and tetragonal phases in FeTe^13,14^. Theoretical calculations have predicted that the hexagonal FeTe is the most thermodynamically favorable phase^16,19,21^. Thus, the growth temperature in the CVD process is essential to realize the phase transition. We found that the phase of FeTe was sensitive to its growth temperature (*T*_growth_). As shown in Fig. 1, at a relatively high temperature, hexagonal FeTe will be the dominant phase. Decreasing *T*_growth_ will lead to the formation of tetragonal FeTe. A clear trend can be seen from the following optical images (Fig. 2a, d; Supplementary Fig. 2a–c). Notably, square-shaped FeTe crystals with an edge size of about 40 μm were obtained at a *T*_growth_ of 530 °C (Fig. 2a). At 550 °C, the obtained nanosheets remain square-like, with slightly increased thickness estimated by the optical contrast (Supplementary Fig. 2a). However, a mixed square and hexagonal shapes were observed at 570 °C with decreased edge length (Supplementary Fig. 2b). When *T*_growth_ was further increased to 590 °C, the resulting FeTe nanoplates exhibited a homogeneous triangular shape with maximum domain sizes exceeding 60 μm (Fig. 2d). Further increasing *T*_growth_ to 610 °C yielded thicker nanoplates again (Supplementary Fig. 2c). It should be pointed out that, the domain sizes and flake thickness of FeTe crystals do not simply increase with temperature. Actually, the obtained FeTe samples by CVD method at different temperatures always show a distribution with different grain size and thickness (Supplementary Fig. 2d, e). The phase evolution of FeTe flakes from tetragonal to hexagonal highly relies on *T*_growth_ (Supplementary Fig. 2f). These results further prove that maintaining a relatively high temperature is essential for obtaining the thermodynamically stable hexagonal FeTe phase. Similarly, the reported literatures have also shown that the temperature and amount of precursor have a key role for phase transformations in 2D materials^22–25^.

### Structural characterization of FeTe crystals

Further characterizations were performed to investigate the morphology and composition of the as-obtained FeTe crystals. The atomic force microscopy (AFM) image in Fig. 2b shows an individual tetragonal FeTe flake with a thickness of 3.6 nm. As for hexagonal FeTe, the thickness can be tailored down to 2.8 nm (Fig. 2e), which is extremely thin for a non-layered material. Figure 2c shows the Raman spectra of the as-grown tetragonal FeTe flakes. Two obvious Raman peaks were located at 130 and 152 cm^−1^, corresponding to the E_g_ and A_1g_ modes of tetragonal FeTe, which are in good accordance with the previous studies^26^. A similar result with different peak positions was also observed in the Raman spectra of the hexagonal FeTe crystal (Fig. 2f). Supplementary Figure 3 shows optical images and corresponding Raman intensity maps for tetragonal and hexagonal FeTe flakes, respectively, suggesting the high crystallinity and uniformity of the FeTe crystals. In addition to Raman characterization, X-ray photoelectron spectroscopy (XPS) was used to analyze the chemical composition of the as-grown FeTe nanosheets. The XPS spectra and analysis (Supplementary Figs. 4 and 5) demonstrate both tetragonal and hexagonal phase FeTe nanosheets are reasonably stoichiometric, without oxidation or any residual chlorine.

**Fig. 3:**
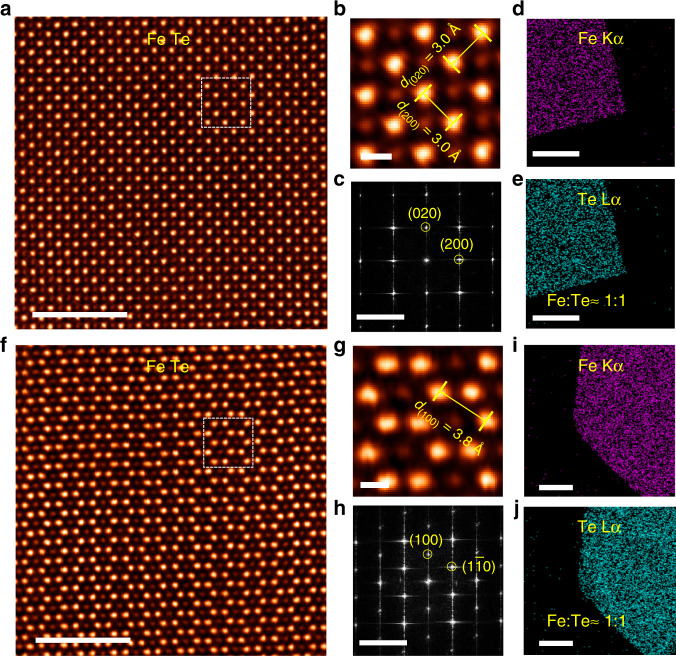
Atomic morphology of tetragonal and hexagonal-shaped FeTe crystals. **a**, **f** Atomic-resolution STEM-ADF images of (**a**) tetragonal, and (**f**) hexagonal-shaped FeTe crystals. **b**, **g** The magnified STEM images from the white box regions in **a** and **f**, respectively. **c**, **h** Corresponding FFT patterns of **a** and **f**, respectively. **d**, **e**, **i**, **j** EDS mapping of **d** and **e** tetragonal, and **i**, **j** hexagonal-shaped FeTe crystals. Scale bars: 5 nm in **a**, **f**; 0.5 nm in **b**, **g**; 5 nm^−1^ in **c**, **h**; 1 μm in **d**, **e** and 500 nm in **i**, **j**.

To probe the atomic structural differences of CVD grown tetragonal and hexagonal-shaped FeTe crystals, aberration-corrected scanning transmission electron microscopy–annular dark field (STEM-ADF) imaging was applied. The image contrast in STEM-ADF image is intimately tied to the *Z* atomic number varying approximately as *Z*^1.6–1.7^, and thereby the Z-contrast STEM image is widely employed to identify the atomic structures in 2D materials^27,28^. The FeTe crystals were transferred from the SiO_2_/Si substrate to TEM grids, as shown in Supplementary Fig. 6, through a surface-energy-assisted transfer protocol^29,30^. The transfer details are provided in the “Methods” section. A typical atomic-resolution STEM-ADF image of the tetragonal shaped FeTe crystal along the [001] zone axis was depicted in Fig. 3a. Following the intuitive STEM image, it can be seen that in line with the macroscopically manifested tetragonal crystal. The tetragonal FeTe crystals comply with a P_4g_ wallpaper group symmetry. The in-plane Te–Te and Fe–Fe bonds are estimated to be ~3.0 Å, as verified by the zoom-in STEM image (Fig. 3b). No discernible extended defects are spotted in the STEM images confirming that the as-grown tetragonal shaped FeTe crystals are highly crystalline. In addition, the corresponding fast Fourier transform (FFT) pattern reveals a singlet set of spots which further corroborate the high crystallinity of the crystal (Fig. 3c). Apparently, it is a typical layered material where the interlayer bonding is raised by weak Te–Te interaction. The Fe and Te elemental distribution as suggested by the energy-dispersive X-ray spectroscopy (EDS) mapping (Fig. 3d, e) is homogenous throughout the entire crystal, and the chemical stoichiometry is calculated as FeTe.

In stark contrast to the tetragonal shaped FeTe crystal, the STEM image (Fig. 3f) of hexagonal-shaped FeTe crystal reveals an in-plane six-fold symmetry along the [001] zone axis. The in-plane Te–Te or Fe–Fe bond is calculated as 3.8 Å according to the enlarged STEM image (Fig. 3g). In parallel, no structural defects or stacking faults are observed throughout the flakes suggesting that the hexagonal-shaped FeTe crystal is highly crystalline. The single crystallinity is further verified by the corresponding hexagonal-shaped FFT pattern (Fig. 3h), where only one set of spots can be observed. Structurally, closed-packed Te atomic planes take periodic ABAB stacking order, and the closed-packed Fe atomic planes alternatively occupy the octahedral vacancies created by the AB-stacked Te atomic planes. In other words, the hexagonal-shaped FeTe takes a periodic ACBC stacking registry, where the small and capital letters represent Fe and Te closed-packed atomic planes, respectively. Hence, the hexagonal-shaped FeTe is no longer a layered material. The chemical composition of hexagonal-shaped FeTe crystal was further analyzed by EDS mapping (Fig. 3i, j). The elemental distribution of Fe and Te elements is uniform and homogeneous. In addition, the chemical stoichiometry of Fe and Te is ~1:1.

### Magnetoelectric characterization of FeTe samples on SiO_2_/Si substrate

**Fig. 4:**
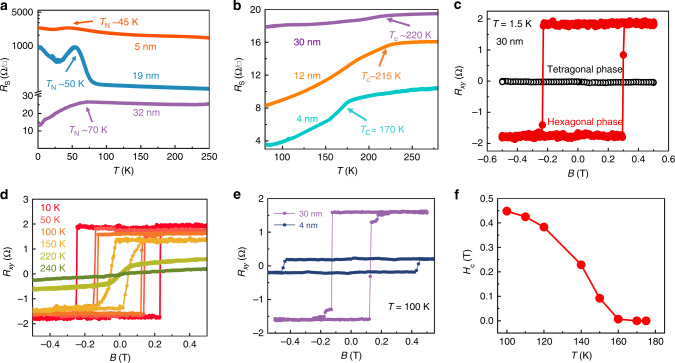
Magnetic characterization of the FeTe nanosheets. **a**, **b** The temperature dependence of the longitudinal sheet resistance (*R*_S_) of tetragonal (**a**) and hexagonal (**b**) FeTe devices with different thicknesses, respectively. **c** The magnetic field dependence of the Hall resistance (*R*_*xy*_) at 1.5 K corresponding to 32 nm tetragonal FeTe and 30 nm hexagonal FeTe. **d** Temperature-dependent anomalous Hall effect (AHE) of the hexagonal FeTe with 30 nm. **e** AHE hysteresis loops of hexagonal FeTe devices with 30 and 4 nm measured at 100 K. **f** The coercive field of 4 nm hexagonal FeTe device as a function of the temperature.

Although magnetic signal can be directly detected by bulk-sensitive techniques, such as vibrating sample magnetometer (VSM) and superconducting quantum interference device (SQUID), magnetoelectric measurement is more suitable to characterize the weak magnetism from low dimensional nanoflakes^2,12^. For instance, bulk-sensitive techniques inevitably detect the signal from magnetic contaminations, because ultrathin samples’ weak magnetic signal is often comparable or even weaker than that of contaminations obtained from various sources, such as substrate, glue, magnetic particles from laboratory environment. Supplementary Fig. 7 shows the optical images of tetragonal and hexagonal FeTe Hall-bar devices with different thicknesses, which were fabricated directly onto the SiO_2_/Si substrates and well-protected by the hexagonal boron nitride (h-BN) capping layer. We should note that tellurides are susceptible to ambient degradation. Generally, our FeTe crystals can survive in the air for only half an hour (Supplementary Fig. 8). So, FeTe samples were always immediately moved to an Ar glove box after the growth process. The glove box (Supplementary Fig. 9) is equipped with an optical microscope with very long-distance objectives, a spin coater, a heating stage and other elements required for the PMMA coating and h-BN encapsulation process. The details are described in the “Methods” section. We reduced the exposure time of FeTe samples in the air as much as possible to avoid the degradation. Figure 4a depicts the temperature-dependent longitudinal sheet resistance (RT) of tetragonal FeTe devices with the thicknesses of 32, 19, and 5 nm, respectively. A clear change of resistance was observed at all three thicknesses, corresponding to the paramagnetic (PM) to AFM phase transition. The *T*_N_ gradually decreases from 70 to 45 K as the thickness decline, which is consistent with its weak interlayer coupling^6^. Both the linear Hall effect (black curve in Fig. 4c) and temperature-dependent magnetic moment results (Supplementary Fig. 10) in the 32 nm tetragonal FeTe device confirmed the AFM behavior with *T*_N_ ~ 70 K, below which the spontaneous magnetization of FeTe lattice exceeds over the thermal fluctuation-induced net magnetic moment^31^.

Figure 4b shows the RT of hexagonal FeTe devices with thicknesses 30, 12, and 4 nm. The phase transition from non-magnetic (NM) to ferromagnetic (FM) phase was observed at a *T*_c_ ~ 220 K for 30 nm, *T*_c_ ~ 215 K for 12 nm and *T*_c_ ~ 170 K for 5 nm, respectively. The ferromagnetism was further confirmed by the Hall measurement. As for the thick hexagonal FeTe (30 nm), the anomalous Hall effect (AHE) observed clearly at 1.5 K within ±0.5 T magnetic field range (red curve in Fig. 4c). With the temperature increases from 1.5 to 220 K, the temperature-dependent saturation moment and the coercive field (*H*_c_) gradually decrease, above which the AHE vanishes (Fig. 4d). As the thickness declined, Fig. 4e presents the typical AHE hysteresis loops for both 30 and 4 nm hexagonal FeTe devices measured at 100 K within ±0.5 T magnetic field range. *H*_c_ develops from 0.13 to 0.44 T while the saturated jump of |∆*R*_*xy*_| suppresses from 1.58 to 0.2 Ω when the thickness decrease from 30 to 4 nm. Supplementary Fig. 11 shows the temperature-dependent AHE hysteresis loops for a 4 nm hexagonal FeTe device. A smaller *H*_c_ with a larger saturated resistance was observed as the temperature gradually rises. As shown in Fig. 4f, the coercive field gradually shrank as the temperature rises and varnished when the temperature is higher than *T*_c_ ~ 170 K, supporting the *T*_c_ of 4 nm hexagonal FeTe device is ~170 K.

### Insight of the origin of magnetic order in FeTe crystals

**Fig. 5:**
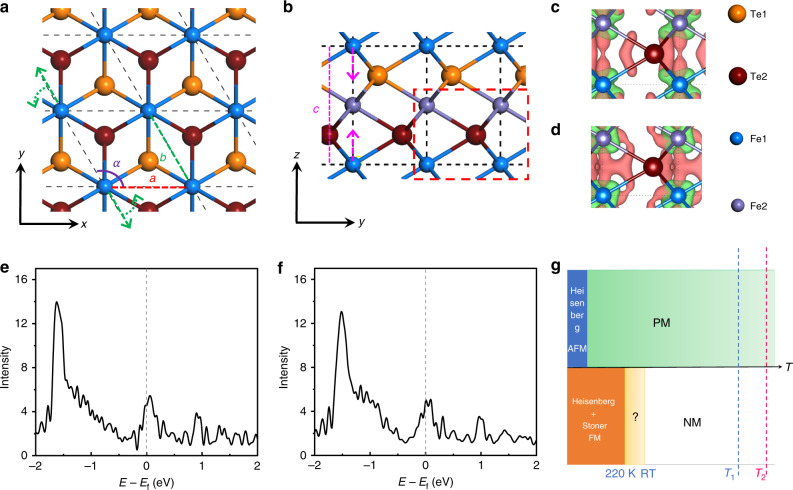
Structure and magnetism of undistorted and distorted hexagonal FeTe. **a** Top view of hexagonal FeTe. Lattice constants *a* and *b* are marked with the red and green dashed lines, respectively, whereas the purple arc represents angle *α* between the two lattice vectors. Green dashed arrows show the directions of lattice reshaping, e.g., extended *b* and enlarged *α*, in the distorted lattice. **b** Side view of hexagonal FeTe. Lattice constant *c* is marked with the pink dashed line while the two pink dashed arrows denote a shrank *c* in the distorted structure. **c**, **d** Atomic differential charge densities of the undistorted and distorted structures in their most stable magnetic orders with an isosurface of 0.006 *e*/Bohr^3^. The plotted region is marked in the red dashed rectangle in **b**. Here, red and green isosurface contours correspond to charge accumulation and reduction after Fe and Te atoms bonding together, respectively. **e**, **f** Total density of states of *d* orbitals for undistorted and distorted hexagonal FeTe, respectively. **g** Phase diagram of magnetism of the tetragonal and hexagonal phases as a function of temperature. The tetragonal (upper) and hexagonal (lower) phases are separated by the lateral axis of temperature. Blue and red dashed lines represent *T*_growth_ of the tetragonal (*T*_1_) and hexagonal (*T*_2_) phases, respectively. Here, the paramagnetic phase is modeled by an Néel AFM configuration for the tetragonal phase above *T*_c_, while it is, most likely, an NM one for the hexagonal phase.

Density-functional theory calculations were carried out to determine the magnetic ground state of the hexagonal FeTe. Schematic top and side views of the hexagonal FeTe structure are shown in Fig. 5a, b. Our non-magnetic calculation reveals lattice constants *a* = 3.90 Å and *c* = 5.36 Å, which are comparable with the STEM (Fig. 3g) and electron diffraction (Supplementary Fig. 12) values of 3.8 and 5.2 Å measured at room temperature, respectively. However, if Fe cations are allowed to have local magnetic moments, lattice constant *a* elongates to roughly 4.2 Å regardless of its long-range magnetic ordering. Together with the *T*_C_ of ~220 K (Fig. 4b), these results suggest the structure observed in STEM is, most likely, a non-magnetic but not a paramagnetic hexagonal FeTe. In terms of the tetragonal phase, however, the measured value of *a* = 3.0 Å is closer to our theoretical value of Néel AFM phase of *a* = 2.90 Å, but much larger than the non-magnetic value of *a* = 2.71 Å. An Néel AFM configuration can mostly simulate properties of the corresponding paramagnetic phase, which implies local magnetic moments of the tetragonal phase persist at room temperature, consistent with previous calculations and experiment measurements^16^. In light of this, we can infer that the tetragonal and hexagonal phases are, most likely, paramagnetic and non-magnetic, respectively at room temperature and during the synthesis process (Fig. 5g).

We first consider a local magnetic moment picture for the ferromagnetism of the hexagonal phase. Significant magnetostriction was observed in the FM–FM (both intra- and interlayer FM, *a* = 4.23 Å, *c* = 5.81 Å) and FM–AFM (intralayer FM and interlayer AFM, *a* = 4.19 Å, *c* = 5.83 Å) configurations, which are yet to be confirmed by low-temperature STEM experiments. Details of all considered magnetic orders are available in Supplementary Fig. 13. Among these configurations, the FM–AFM order (Fig. 5c) is, however, over 20 meV/Fe more favored than other orders for a perfect hexagonal FeTe (Supplementary Table 1). A structural distortion further lowers the energies of all magnetic orders (on the order of 100 meV/Fe). As a result of the distortion, lattice constant *c* shows a considerable shrink from roughly 5.8 to 5.45 Å (Fig. 5b), associated with a stretched lattice constant *b* (from 4.19 to 4.29 Å) and an increased angle *α* (from 120.0° to 121.4°) (Fig. 5a). The broken in-plane three-fold symmetry aside, the distorted structure primarily leads to a much shorter interlayer Fe–Fe distance, from 2.9 to 2.73 Å, and thus enhances their FM direct exchange through the formed interlayer Fe–Fe bonding. Such enhanced direct exchange favors the FM–FM order, with strongly enhanced FM *J*_1_ and *J*_3_ (Supplementary Fig. 14 and Supplementary Table 2), in a slightly distorted structure (Fig. 5d; Supplementary Table 1). As a result, the FM–FM order is over 7 meV/Fe more favored than other magnetic configurations in the distorted structure (Supplementary Table 1). The comparison of intact and distorted lattices suggests an energetic competition between the interlayer Fe–Fe bonding and the lifted energy in the distorted lattice, which are associated with the change of interlayer magnetism. This thus implies a likely magnetoelastic effect, up to 1% for in-plane and 6% for out-of-plane, potentially observable in the hexagonal FeTe under certain conditions of temperature, external strain and magnetic field. These results also indicate that the structural distortion might have a paramount role in the observed ferromagnetism.

Spin-exchange parameters for both the undistorted and distorted structures are listed in Supplementary Fig. 14 and Supplementary Table 2, which predict a *T*_c_ value around 290 K using Metropolis Monte Carlo (MC) simulation in a 3-layer 3D Ising lattice. Given the fact that MC simulation of the Ising model usually overestimates *T*_c_ two or three times^32^, the hexagonal phase is expected to have a *T*_c_ value around 100–150 K in consideration of the Heisenberg local exchange picture, substantially lower than the measured value of ~220 K. This low *T*_c_ value indicates another mechanism may corporately result in such a high *T*_c_ value of 220 K. Stoner ferromagnetism mechanism is another likely reason for the FM. Non-magnetic calculations were performed to calculate the DOSs using the FM relaxed geometries. Figure 5e, f shows the plots of the total *d*-orbital DOSs of the undistorted and distorted structures, respectively, both of which meet the Stoner criterion, i.e., (*U*′/*N*)*ρ*(*E*_df_) > 1, compellingly indicating the Stoner ferromagnetism in this metallic system. In addition, the distorted structure shows a larger (*U*′/*N*)*ρ*(*E*_df_) value (1.95) than that of the undistorted structure (1.55), evidencing a stronger Stoner ferromagnetism in distorted FeTe (see Supplementary Fig. 15 for details). The Stoner FM could also explain the coexistence of the high *T*_c_ of 220 K and the non-magnetic state observed at RT. In summary of these results, we conclude that the hexagonal phase exhibits a Heisenberg plus Stoner FM below 220 K, NM above RT and, most likely, NM between 220 K and RT, as illustrated in Fig. 5g.

## Discussion

Vibrational frequency calculations were performed to compare free energies of different phases. The free energy plot (Supplementary Fig. 16) shows, however, no thermally driving phase transition occurred from 0 to 1000 K for the bulk phases. In light of this, we believe the controlling of phases is a result of either kinetic reasons or thermally driven transitions at the thin-film limit. Our results of surface-energy calculation (Supplementary Fig. 17) show ultra-low surface energy (1.9 meV Å^−2^) of the Te-terminated hexagonal slab in a Te-rich condition, roughly an order of magnitude smaller than those of other slabs. Thus, the Te-terminated hexagonal slab is the most energetically favored one among all hexagonal and tetragonal slabs in the Te-rich growth condition that could be reached by increasing the Te source temperature, as we demonstrated in our experiments. In addition, our vibrational frequency calculations indicate that Te-terminated hexagonal FeTe mono- and few-layers are, exceptionally, unstable and tend to transform into Fe–Te clusters at 0 K, with imaginary frequencies of e.g., 79.21 and 188.83 *i* cm^−1^ for the monolayer. Given the experimentally synthesized hexagonal phase, we infer that the few-layers hexagonal FeTe is most likely, stabilized by non-harmonic effects, which suppresses the phonon softening and is more pronounced at high temperatures^33^. This partially explains the reason why the hexagonal phase is a high temperature phase.

In conclusion, a facile and effective strategy for growing ultrathin 2D magnetic FeTe crystals with tunable structural phases was developed. We can selectively obtain layered tetragonal FeTe nanoplates and non-layered hexagonal FeTe nanoplates as square or trigonal geometries, with thickness down to 2.8 and 3.6 nm, respectively. Systematic RM, AFM and STEM studies revealed fine structural information of these FeTe flakes and confirmed they were single crystals with high crystallinity. Furthermore, the magneto transport measurements showed tetragonal FeTe crystal was antiferromagnetic with *T*_N_ decreased as the thickness decline. While the hexagonal FeTe flakes exhibited obvious ferromagnetic properties with a thickness-dependent transition temperature from 220 to 170 K. Our data confirmed the AFM and FM order for tetragonal and hexagonal FeTe flakes down to 5 and 4 nm, respectively. Detailed calculated results indicated a structural distortion in hexagonal FeTe is responsible for the observed ferromagnetism. Our work paves the way for controlled growth of other 2D magnetic materials by the CVD method and provides a tunable material system for investigating 2D magnetism and potential applications in future spintronic devices.

## Methods

### Atomically thin FeTe crystals synthesis

2D polymorphous FeTe flakes were synthesized on SiO_2_/Si substrates by an ambient pressure CVD method. The reaction process was conducted in a 1.2-m length, 2-inch outer diameter quartz tube heated by a three-zone furnace (Thermcraft (XST-3-0-18-2V2)), which had three independently controlled temperature zones. Te powder (99.997%, Sigma Aldrich) and ferrous chloride (FeCl_2_, 99.9%, Sigma Aldrich) were used as solid precursors and reactants. The Te powder (in a quartz boat) was placed near to the gas inlet in the first heating zone while the ferrous chloride powder, loaded into the third heating zone, was placed in an alumina boat covered with an inclined downward-facing SiO_2_/Si substrate. The second heating zone was kept at the same condition as that of the first heating zone. Prior to the growth process, the furnace was purged by the flowing of 300 sccm high-purity Ar gas for 5 min. Then, 100 sccm Ar and 10 sccm H_2_ mixtures were introduced into the CVD system. The tellurium was heated to 520 °C in 20 min while the growth zone of ferrous chloride was gradually increased to different temperatures (520–630 °C) in 20 min. Set temperature for all the heating zones was kept for another 10 min for growth of temperature. After synthesis, the furnace was rapidly cooled down to room temperature with the assistance of electric fans. Obvious etching phenomena were observed when the furnace was naturally cooled without any external perturbations for both tetragonal and hexagonal FeTe flakes (Supplementary Fig. 18).

### Preparation of STEM sample

The transfer of the FeTe flakes from SiO_2_/Si substrate to TEM grids followed a surface-energy-assisted transfer approach^29,30^, which was shown in (Supplementary Fig. 19). Firstly, the FeTe grown on the SiO_2_/Si substrate coated with PMMA film was cut into a small size piece (<2 mm × 2 mm), which floated on the surface of 1 mol L^−1^ KOH solution due to the surface tension of water (Supplementary Fig. 19a). A fine point tweezer press the substrate gently to let some water insert into the PMMA/silicon interface quickly because of the hydrophobic nature of PMMA. In only tens of seconds, we can see the separation of PMMA and silicon substrate at the edge (Supplementary Fig. 19b). Then, we use the tweezer grasp part of the separated film and peel off the whole film from the substrate (Supplementary Fig. 19c). In fact, some PMMA/FeTe film was scraped from the substrate directly, which did not contact with the KOH solution. These FeTe flakes without oxidation are suitable for the STEM characterization. We could pick up the PMMA/FeTe assembly with a tweezer and attached it to a TEM grid (Supplementary Fig. 19d). Finally, PMMA was removed by acetone. The whole transfer process was performed in an Ar glove box and took about 5 min, which indeed caused the increase of H_2_O content from a normal value (below 1 ppm) to around 20 ppm. However, the entire process only lasts for a few minutes. And we kept the open of cleaning gear button for the glove box during the transfer process. Thus, we can work with aqueous solutions for a short time inside a glove box.

### Devices fabrication and transport measurement

The tetragonal and hexagonal FeTe flakes grown on SiO_2_/Si substrate were quickly moved to an Ar glove box and spin coated with 950 K PMMA (MicroChem) at 3000 r.p.m. for 1 min. After leaving the PMMA layer consolidating on a 120 °C hotplate for 5 min in the Ar glove box, small markers were fabricated using standard e-beam lithography (Nova nanoSEM 230 with digital pattern generator Nabity-nanometer pattern generation system (NPGS)) near the identified sample for subsequent fabrication of Hall-bar devices. The Ti/Au (5/70 nm) electrodes were deposited using an electron-beam evaporator (Kurt J. Lesker Nano 36 Thermal Evaporator) followed by lift-off in acetone. The electrical contacts were made by wire bonding Al wires onto the six electrodes on a Hall-bar structure. Transport experiments were performed in an Oxford cryostat with the temperature ranging from 300 to 2 K and the magnetic field up to 8 T. The sample resistance and Hall effect were measured by Keithley 6221 triggered with Keithley 2182 with the 21 Hz frequency.

### Characterizations of 2D FeTe crystals

The as-obtained FeTe nanoplatelets were further characterized by optical microscopy (Olympus BX53M), RM (WITEC alpha 300R Confocal Raman system using a 532 nm laser as the excitation source), AFM (Asylum Research Cypher Scanning Probe Microscope system with a tapping mode), and XPS (Kratos AXIS Supra spectrometer with a monochromatic Al K-alpha source). ADF-STEM imaging was conducted on an aberration-corrected JEOL ARM-200F equipped with a cold field emission gun, operating at 80 kV, and an Advanced STEM Corrector (ASCOR) probe corrector.

### Details of DFT calculation

Our density-functional theory calculations were performed using the generalized gradient approximation and the projector augmented wave method^34,35^ as implemented in the Vienna ab-initio simulation package (VASP)^36,37^. The Perdew–Burke–Ernzerhof (PBE)^38^ functional with the density-dependent dispersion correction (dDsC)^39,40^ was adopted for all calculations. A uniform Monkhorst–Pack *k*-mesh of 21 × 21 × 13 was adopted for sampling over the Brillouin zone of hexagonal FeTe and a 20 × 20 × 8 mesh for the tetragonal phase. A plane-wave kinetic energy cutoff of 700 eV was used for structural relaxation while 400 eV for energy comparison. All atoms were allowed to relax until the residual force per atom was less than 0.01 eV Å^−1^. Full relaxation of all atomic coordinates using the dispersion-corrected PBE functional well addresses the Te-height-dependent magnetic ground state issue^41^, and finds the correct magnetic ground state for tetragonal FeTe^16^. We also consider spin-orbit coupling (SOC) for energy comparisons among different magnetic configurations. A 2 × 2√3 × 1 orthorhombic supercell, with a *k*-mesh of 4 × 4 × 6, was used to model different magnetic orders in FeTe. An on-site Coulomb interaction energy to Fe *d* orbitals were self-consistently calculated using a linear response method^42^, which revealed *U* = 3.3 eV and *J* = 0.5 eV.

### Derivation of spin-exchange parameters

A Heisenberg model was used to model the magnetism of the bulk FeTe crystal, $H = H_0 - \mathop {\sum}\nolimits_{k = 1}^5 {J_k} \mathop {\sum}\nolimits_{i,j} {\overrightarrow {S_i} \cdot \overrightarrow {S_j} }$, which includes two in-plane spin-exchange interactions, *J*_1_ and *J*_2_ and three interlayer ones, *J*_3_ to *J*_5_, respectively, as displayed in Supplementary Fig. 12. Metropolis Monte Carlo simulation was used to estimate the *T*_c_ value in a three-layer lattice using a 3D Ising model, $H = - \mathop {\sum}\nolimits_{k = 1}^5 {J_k} \mathop {\sum}\nolimits_{i,j} {S_i \cdot S_j}$, in which *S* = 2. A 20 × 20 × 3 lattice was used with a periodic boundary condition.

### Estimation of Stoner criterion

Structural relaxation of non-magnetic configuration does not show structural distortion. We thus kept the in-plane constants *a* = 3.90 Å and *b* = 3.82 Å to calculate the DOS of the distorted structure. We speculate that no structural distortion is acquired at room temperature, since both geometric relaxation and STEM show no significant distortion. What we show in (b) only tends to discuss how distortion affects the DOS. Stoner parameter is defined as *ST* = (*U*′/*N*)*ρ*(*E*_*df*_). Here, $U^\prime = \left\langle {{\it{\epsilon }}_k} \right\rangle {\mathrm{/}}m_d$, where *N* is the number of unit magnetism cells, which equals to 2 in this situation and *ρ*(*E*_*df*_) is the total density of states of all *d* orbitals at the Fermi level for hexagonal FeTe (i.e., 4.54 and 4.93 states/eV/atom for undistorted and distorted structures). $\left\langle {{\it{\epsilon }}_k} \right\rangle$ is the average value of the splitting between the corresponding spin-up and spin-down bands at six high symmetry points *Γ*MKZAR in the BZ, which could be derived from the band structure plotted in Supplementary Fig. 15. *m*_*d*_ is the average value of the magnetic moments on Fe atoms, i.e., 3.4 and 2.9 *μ*_B_ for undistorted and distorted FeTe.

## Supplementary information

Supplementary Information

## Data Availability

The data that support the plots within this paper and other findings of this study are available from the corresponding authors upon reasonable request.
