# Supplementary material for: Phase-controllable growth of ultrathin 2D magnetic FeTe crystals
Source: Nat Commun. 2020 Jul 24;11:3729. doi: 10.1038/s41467-020-17253-x (PMC7382463; doi:10.1038/s41467-020-17253-x)
Supplement: Supplementary file 1 — Supplementary Information [file 41467_2020_17253_MOESM1_ESM.pdf]

Supplementary Information

Phase-controllable growth of ultrathin 2D magnetic FeTe crystals

Kang *et al*.

Supplementary Figures

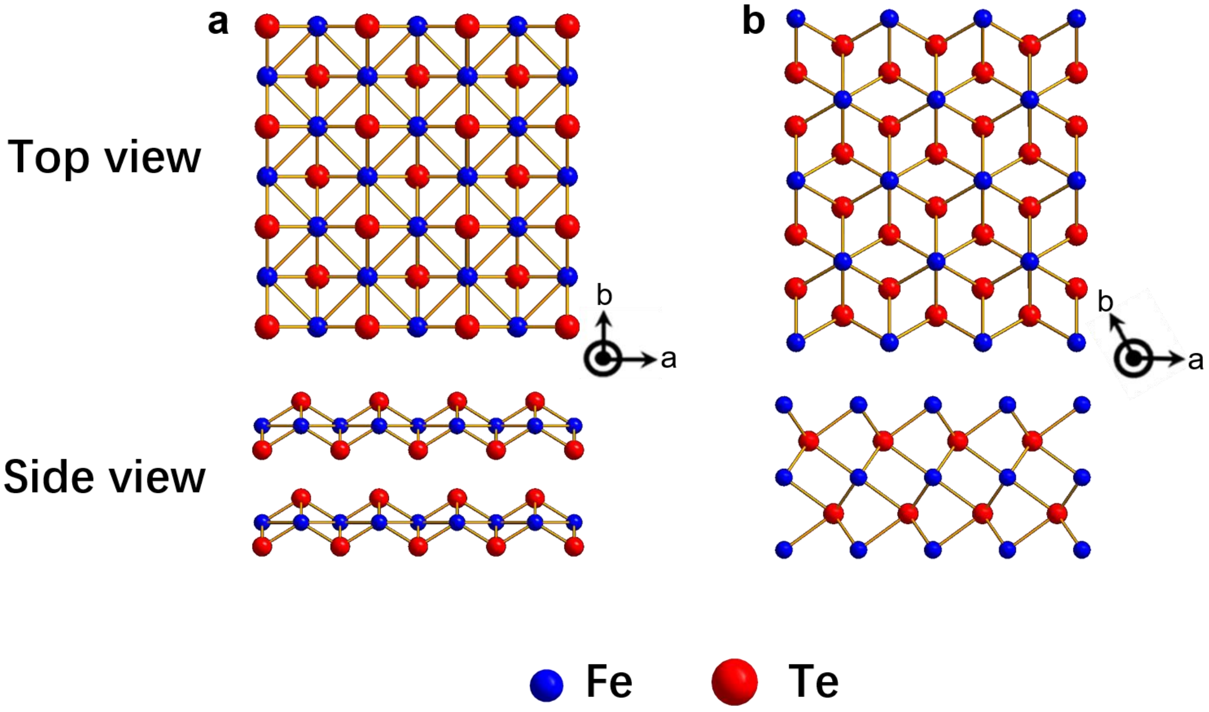

Supplementary Figure 1. Crystal structures of tetragonal (a) and hexagonal (d) FeTe crystals in different sectional views.

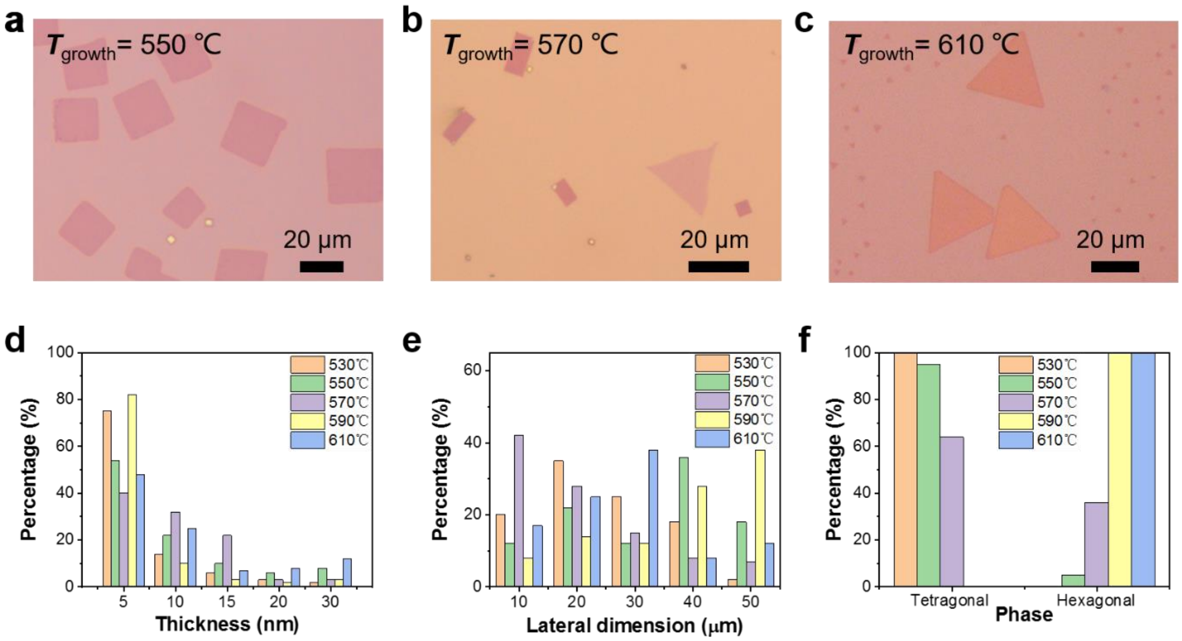

Supplementary Figure 2. a-c Representative morphologies of FeTe crystals synthesized with T_{growth} setting at different temperatures. **d-f** Evolution of thickness (d), grain size (e) and phase structure (f) at different growth temperatures.

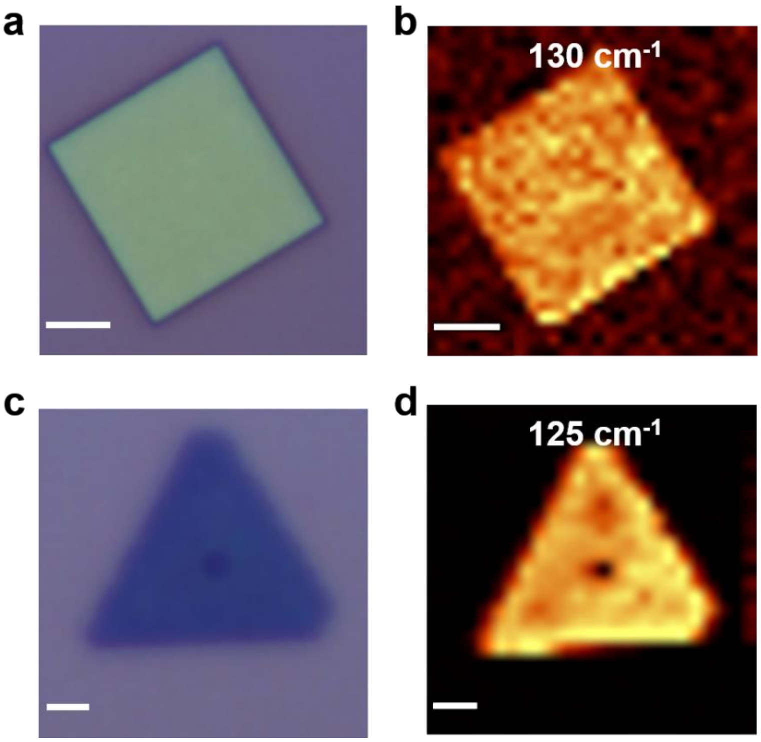

Supplementary Figure 3. a, c Typical optical images of as-grown tetragonal (a) and hexagonal (c) FeTe nanoplatelet on SiO_2/Si substrates. **b, d** The corresponding Raman mappings, respectively. Scale bars: 2 μm in a-d.

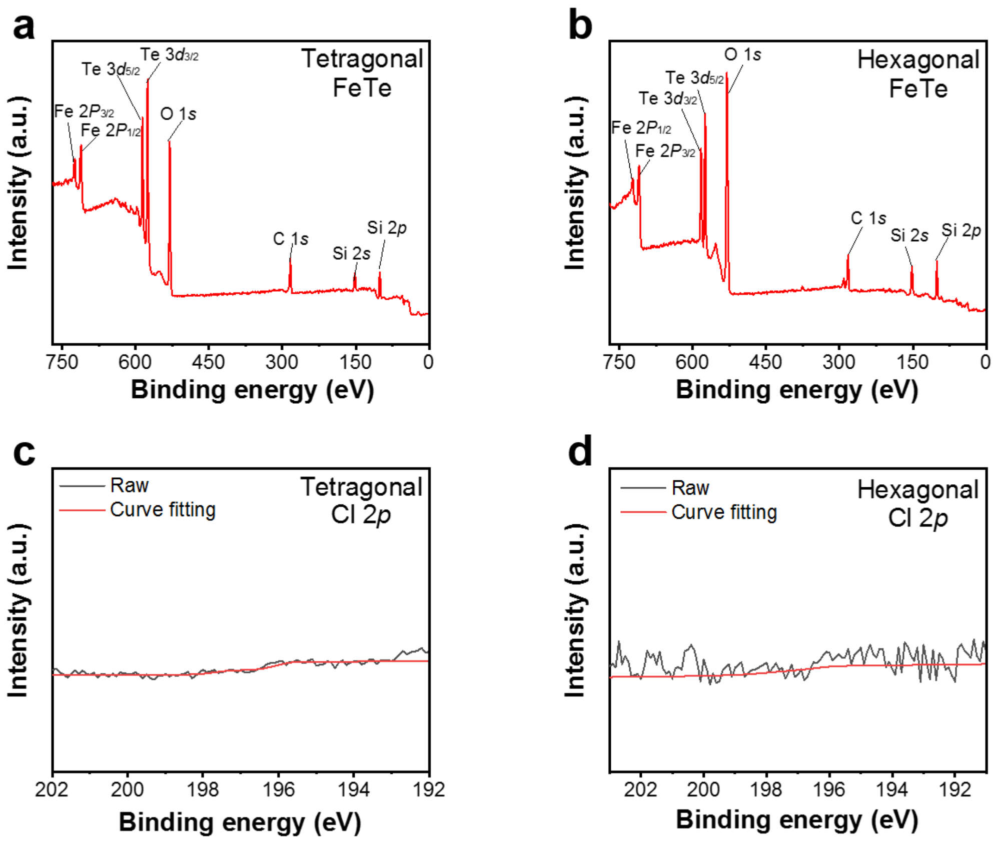

Supplementary Figure 4. a-b XPS full spectra of tetragonal (**a**) and hexagonal (**b**) shaped FeTe crystals. **c-d** Corresponding high-resolution XPS spectra of Cl 2*p*, respectively.

As shown in Supplementary Figure 4a-b, the predominant signal of Fe, Te, O, C and Si can be clearly seen. The peaks of Fe and Te are from the sample, while the signals of O, C and Si come from the charge reference and the SiO_2 substrate. However, there is no Cl signal in the XPS full spectra. We further did the high-resolution XPS scans of the Cl 2*p* region (from 202 eV to 192 eV), which were shown in Supplementary Figure 4c-d. There were also no peaks in the Cl 2*p* region. Depending on the above XPS analysis, it clearly indicated the absence of chlorine residues.

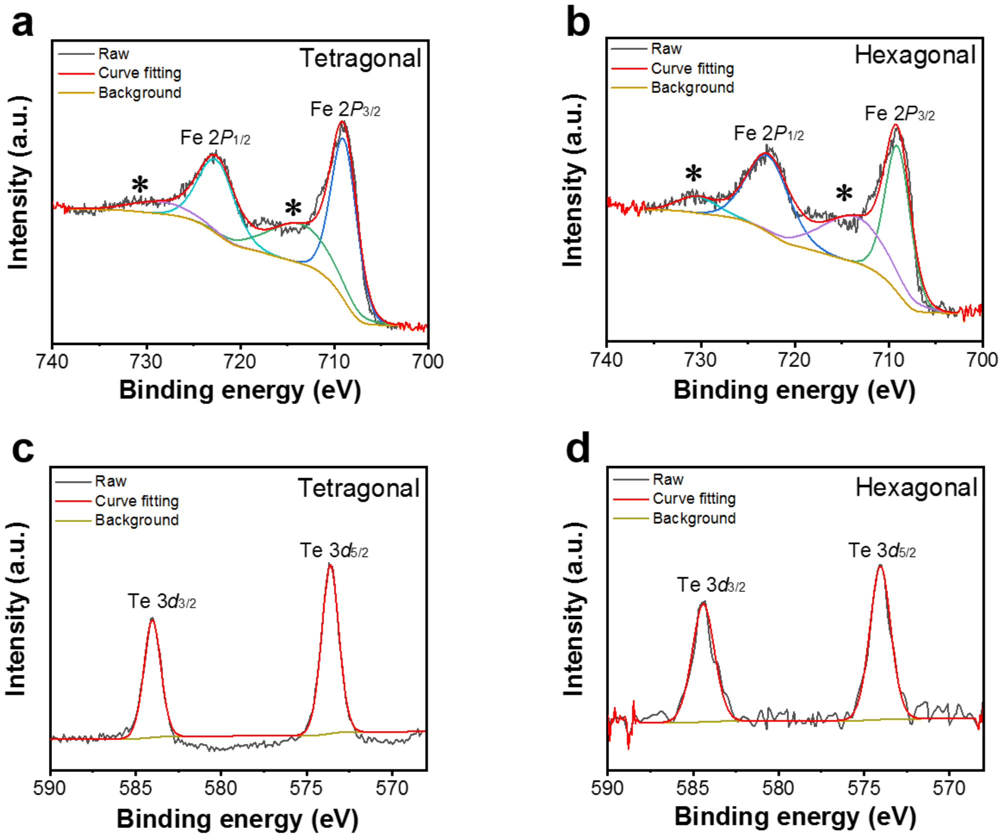

Supplementary Figure 5. High-resolution XPS spectra for obtained tetragonal and hexagonal phase FeTe nanosheets. **a-b** Fe $2P_{1/2}$ and Fe $2P_{3/2}$. The peaks marked by * are satellite peaks. **c-d** Te $3d_{3/2}$ and Te $3d_{5/2}$.

As for the tetragonal shaped FeTe crystal, two doublets of Fe $2P_{1/2}$ and Fe $2P_{3/2}$ are present in the Fe 2*p* spectra (Supplementary Figure 5 a), the first pair occurred at 722.5 and 708.9 eV, while the second pair located at 728.2 and 713.5 eV attributed to satellite peaks, as the previous literature[1]. In the Te 3*d* spectra (Supplementary Figure 5c), the peaks located at around 584 and 573.6 eV represent the Te $3d_{3/2}$ and Te $3d_{5/2}$, respectively. On top of that, the atomic ratio of Fe:Te was calculated to be 1:1.03 through the computation of peak areas and atomic sensitivity factors, indicating the CVD grown tetragonal phase FeTe is reasonably stoichiometric.

Compared with tetragonal shaped FeTe crystal, Fe 2*p* and Te 3*d* XPS spectra for the hexagonal phase FeTe flakes show similar results with different peak positions (Supplementary Figure 5 b and d). The Fe 2*p* spectra (Supplementary Figure 5b) also have two peaks located at 723.1 eV and 709.1 eV, corresponding to the Fe $2P_{1/2}$ and Fe $2P_{3/2}$. The other two peaks at around 730.5 eV and

714.2 eV are the satellite peaks. While the Te 3*d* spectra (Supplementary Figure 5d) has two peaks at around 584.4 and 574 eV represent to the Te $3d_{3/2}$ and Te $3d_{5/2}$. The Fe:Te atomic ratio calculated from the XPS data is 1:0.98, close to 1:1, confirming the formation of stoichiometric hexagonal phase FeTe.

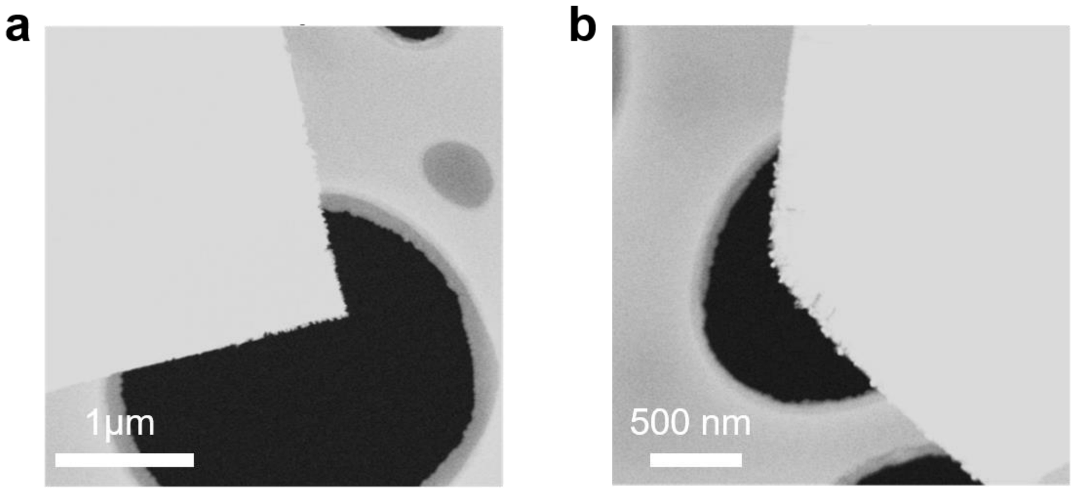

Supplementary Figure 6. Low-magnified STEM images of (a) tetragonal and (b) hexagonal FeTe flakes.

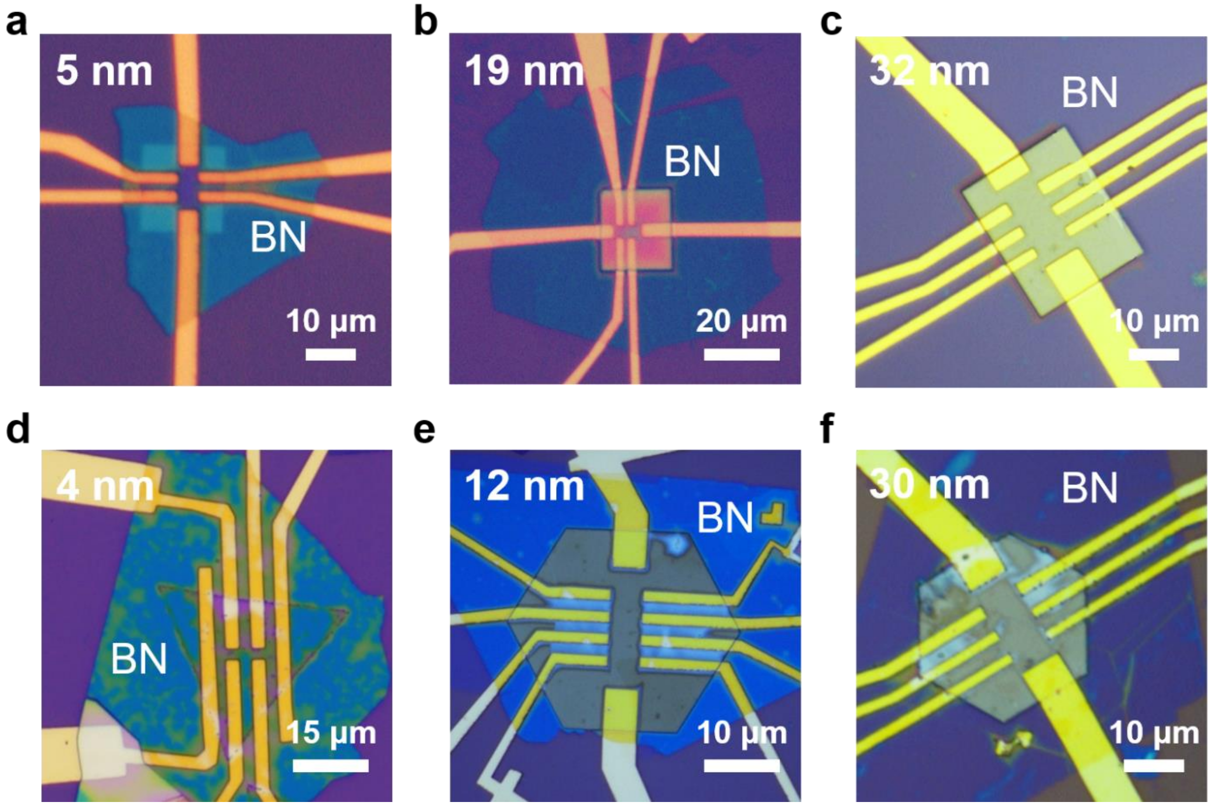

Supplementary Figure 7. a-f Optical images of Hall-bar devices of tetragonal FeTe (**a-c**) with the thickness of 5, 19, 32 nm and hexagonal FeTe (**d-f**) FeTe with the thicknesses of 4, 12 and 30 nm, which are protected by the thin h-BN layers.

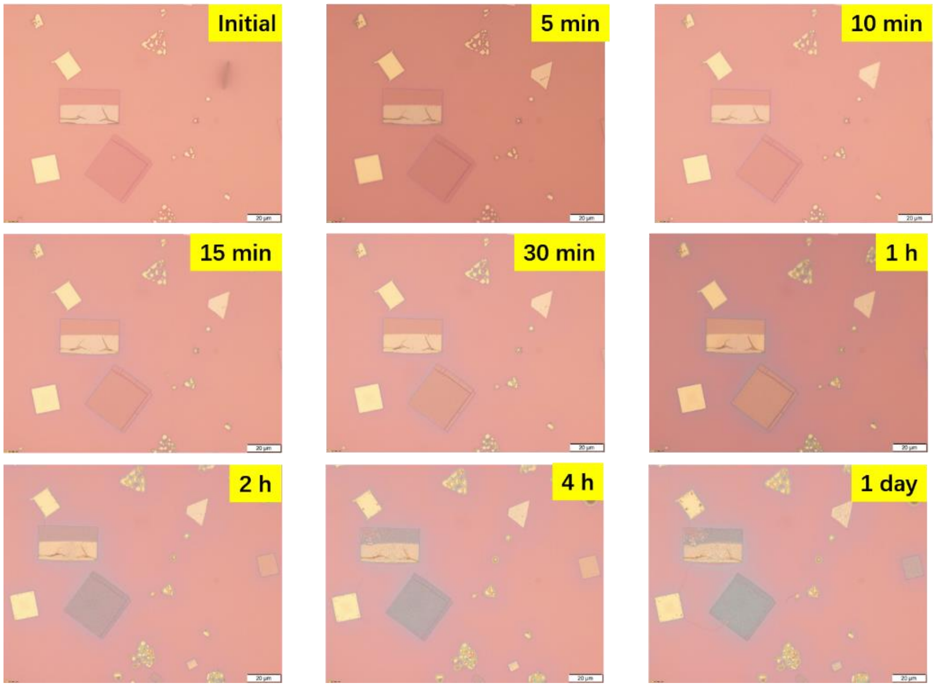

Supplementary Figure 8. Optical images of a mixed square and hexagonal shaped FeTe nanoflakes exposed to the air with the extension of time (from the initial state to 1 day). In the initial 30 min, there is no obvious change according to the optical images. This means our FeTe crystals should be stable in the air for half an hour.

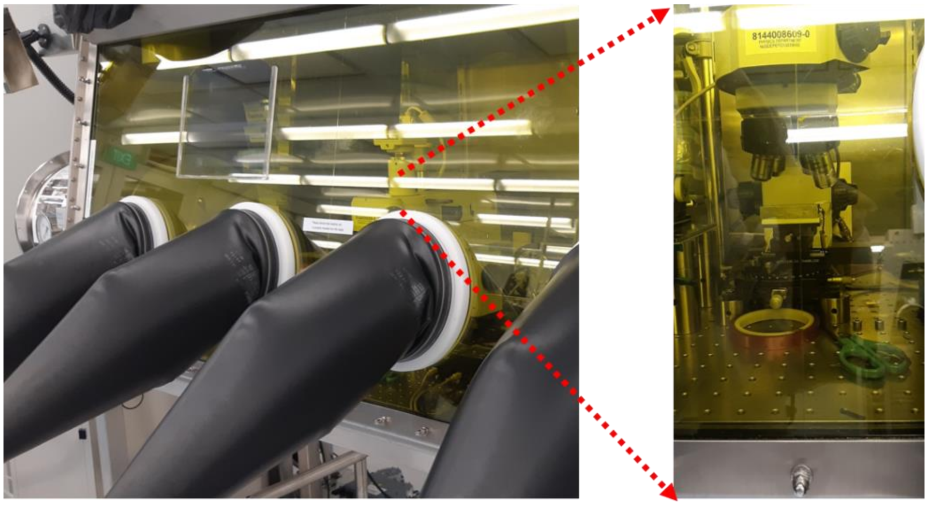

Supplementary Figure 9. Photograph of the Ar glove box. The red arrow aimed at an optical microscope with very long-distance objectives (5x, 20x, 50x, 100x).

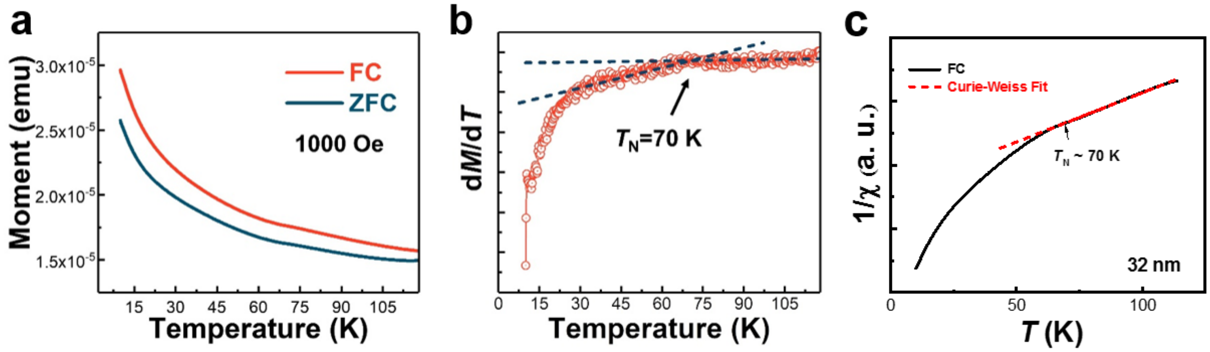

Supplementary Figure 10. Magnetic measurement on the 32 nm tetragonal FeTe flakes. **a** Temperature-dependent magnetic moment as a function of temperature (*M*-*T*) with the field cooling (FC) and zero-field cooling (ZFC). The applied magnetic field is 1000 Oe along the out-of-plane direction. **b** Differential *M*-*T* curve as a function of temperature. **c** The reciprocal of magnetic susceptibility as a function of temperature during the FC process. The data fitting follows the Curie-Weiss law.

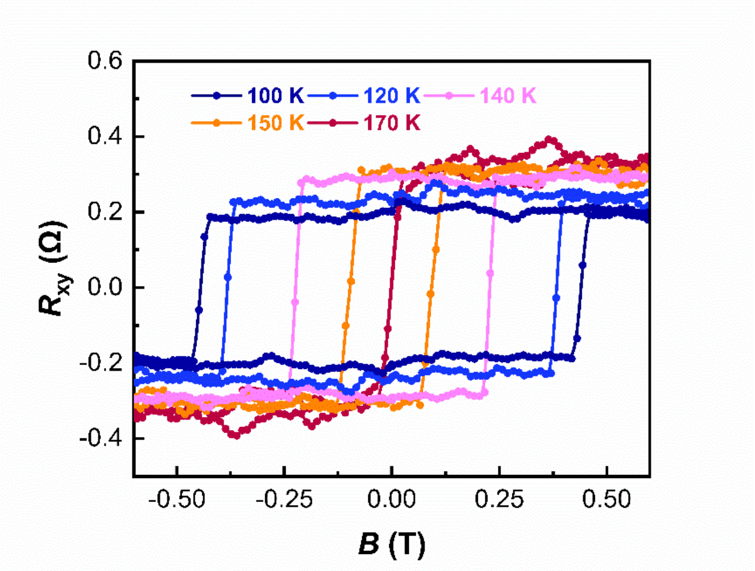

Supplementary Figure 11. The temperature-dependent AHE hysteresis loops of 4 nm FeTe device.

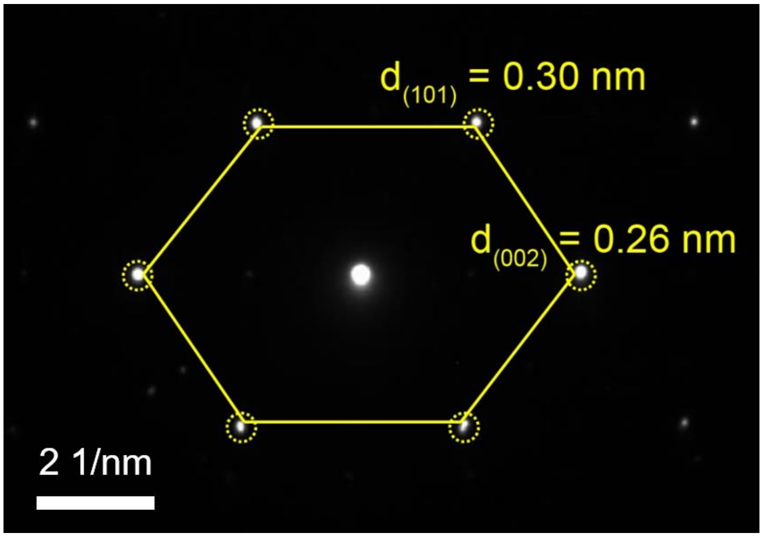

Supplementary Figure 12. The corresponding selected area electron diffraction (SAED) pattern of hexagonal FeTe along the [100] zone axis. The corresponding d-spacing of (101) and (002) are 3.0 Å and 2.6 Å, respectively.

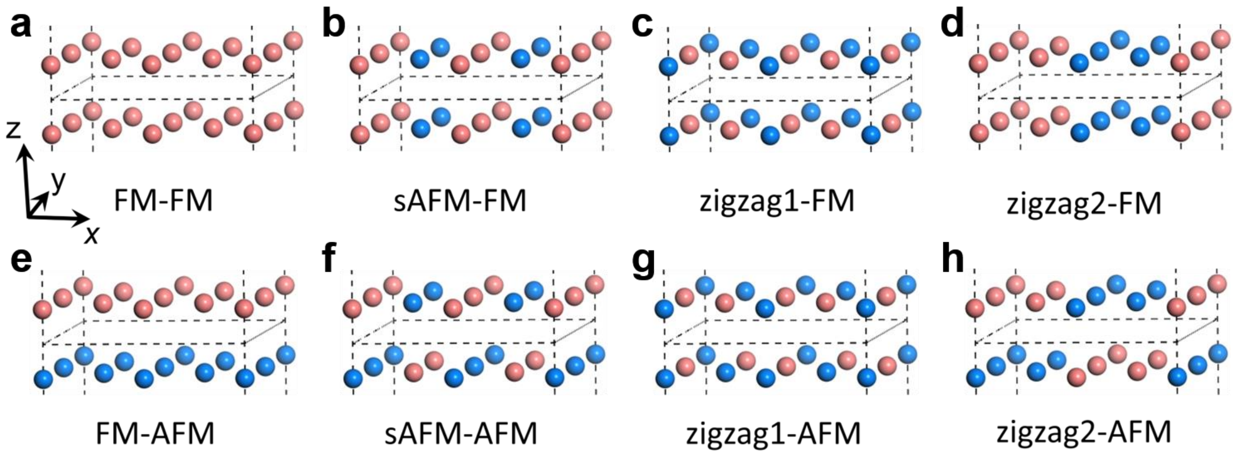

Supplementary Figure 13. Different magnetic order of bulk FeTe. **a-h** Schematic representation of eight magnetic order in FeTe bulk, named FM-FM, sAFM-FM, zigzag1-FM, zigzag2-FM, FM-FM, sAFM-AFM, zigzag1-AFM and zigzag2-AFM respectively. The light coral and royal blue balls represent magnetic moment up and down on Fe atoms, respectively. A $2 \times 2\sqrt{3} \times 1$ orthorhombic supercell was used to show different magnetic order.

Supplementary Table 1. Relative energy between different magnetic orders and the magnetic ground state of the undistorted and distorted structures. Spin-orbit coupling was also considered with the initial magnetic moments parallel to the x, y and z-axis. Given the fact that different magnetic orders might take different easy axis, we thus compared relative energies of those magnetic orders with their magnetic moments paralleled to their easy-axis. The total energies of FM-FM orders in the distorted structure were used as the references. Relative energies were listed in the table to standout energy differences induced by varying magnetic orders (Supplementary Table 1a). Magnetic anisotropic energies (MAE) are also listed in the table (Supplementary Table 1b). To exclude the energy differences from the structure, all the energies were calculated using the structure of FM-FM order for undistorted and distorted systems. Perdew-Burke-Ernzerhof (PBE) functional was used for energy calculations.

a

**FeTe**	Rel_E_w/o SOC (meV/Fe)		Rel_E_SOC (meV/Fe)	
	undistorted	distorted	undistorted	distorted
FM-FM	152.4	**0.0**	149.2	**0.0**
FM-AFM	107.9	11.0	114.9	17.5
sAFM-FM	143.8	16.9	148.4	16.1
sAFM-AFM	160.8	69.5	165.6	68.6
zigzag1-FM	159.2	7.8	154.9	7.3
zigzag2-FM	156.2	6.9	161.6	5.3
zigzag1-AFM	128.6	49.9	134.9	56.9
zigzag2-AFM	169.5	72.8	175.5	72.5

b

**FeTe**	Rel_E_MAE (meV/Fe)					
	undistorted			distorted		
	x	y	z	x	y	z
FM-FM	0.6	**0.0**	1.4	**0.0**	0.6	0.8
FM-AFM	0.0	**0.0**	0.6	**0.0**	0.6	0.7
sAFM-FM	0.4	**0.0**	0.8	0.6	**0.0**	1.5
sAFM-AFM	0.0	**0.0**	0.1	**0.0**	0.0	0.3
zigzag1-FM	1.3	**0.0**	1.4	**0.0**	0.9	1.3
zigzag2-FM	0.0	**0.0**	0.6	0.7	**0.0**	1.4
zigzag1-AFM	**0.0**	0.3	0.3	**0.0**	0.1	0.3
zigzag2-AFM	0.2	**0.0**	0.5	**0.0**	0.5	0.5

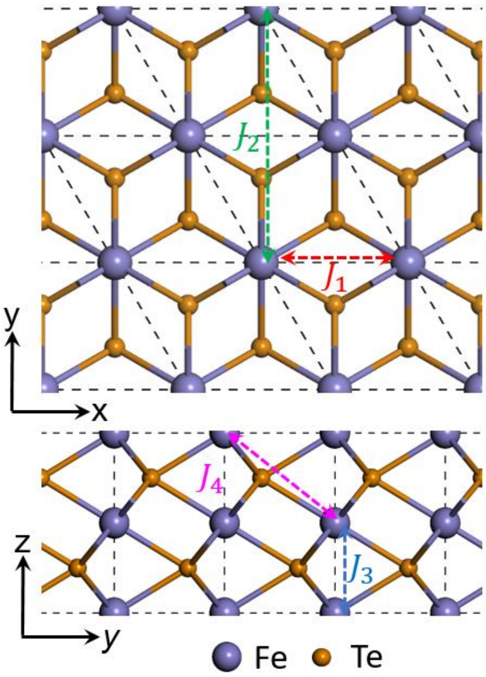

Supplementary Figure 14. Sketch view of spin-exchange parameters of hexagonal FeTe. We used four spin-exchange parameters to describe the exchange interactions in the undistorted and

distorted hexagonal phase. Energies E1 to E6 were used to derive those four parameters, which represent the total energies of FM-FM, FM-AFM, sAFM-FM, sAFM-AFM and zigzag1-FM, respectively (see Supplementary Fig.15 for details). The magnetic configurations in a magnetic unit cell read as follows where N represents the number of unpaired spins on each Fe atom, which is chosen as 4 in our calculations.

$$E_1 = E_0 - \frac{N^2}{4} \times \frac{1}{2}(6J_1 + 6J_2 + 2J_3 + 12J_4) \quad (1)$$

$$E_2 = E_0 - \frac{N^2}{4} \times \frac{1}{2}(6J_1 + 6J_2 - 2J_3 - 12J_4) \quad (2)$$

$$E_3 = E_0 - \frac{N^2}{4} \times \frac{1}{2}(-2J_1 + 6J_2 + 2J_3 - 4J_4) \quad (3)$$

$$E_4 = E_0 - \frac{N^2}{4} \times \frac{1}{2}(-2J_1 + 6J_2 - 2J_3 + 4J_4) \quad (4)$$

$$E_5 = E_0 - \frac{N^2}{4} \times \frac{1}{2}(2J_1 - 2J_2 + 2J_3 + 4J_4) \quad (5)$$

Supplementary Table 2. Spin-exchange parameters and corresponding magnetic moments of Fe cation in undistorted and distorted hexagonal FeTe. Structural distortion leads to ferromagnetic J1 and J3, resulting in a FM-FM order FeTe in a Heisenberg exchange picture at extremely low temperatures. The formed interlayer Fe-Fe bonding lifts some occupied spin-up states at the Gamma and Z points over the Fermi energy. Those states provide local magnetic moments, lifting of which above the Fermi energy thus reduces the local magnetic moment from 3.4 to 2.9 μB in the distorted structure. In addition, such interlayer Fe-Fe bonding offers accumulated electron density between two Fe atoms (Fig. 5c and 5d). The accumulated electron density perturbs the originally symmetrically distributed charge density and thus the geometric

structure of the Fe-Te octahedron, which, therefore, breaks the three-fold symmetry of in-plane Fe-Fe distances, giving rise to a pair of split lattice constants a and b.

	Exchange parameters (meV/f.u.)				Mag.moment(μ_B)
	J_1	J_2	J_3	J_4	
undistorted	-0.9	-1.5	-2.1	-2.1	3.4
distorted	2.6	-0.2	4.9	-0.7	2.9

*Positive and negative J stands for FM and AFM coupling, respectively

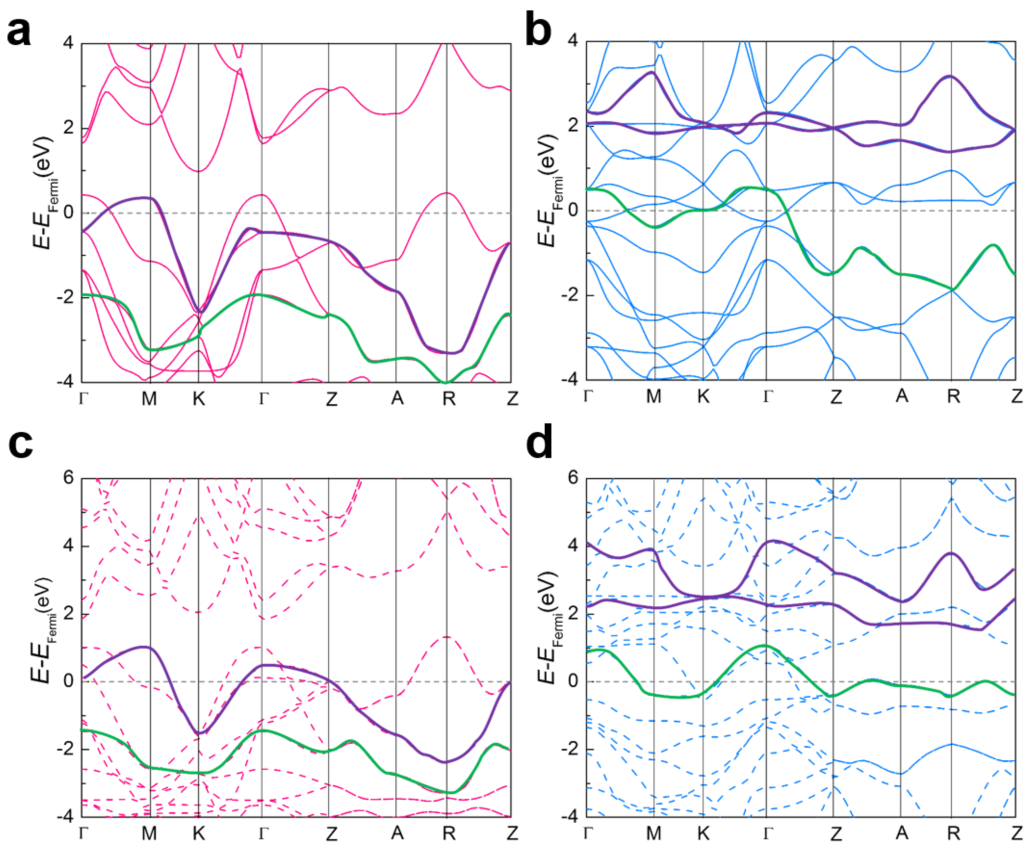

Supplementary Figure 15. Band splitting and the Stoner criterion. **a-b** display the bandstructure of spin-up and spin-down electrons in undistorted FeTe while c-d display those in distorted FeTe. To simplify the problem, we choose representative bands around the Fermi level *i.e*. the purple bands representing the d_{z2} and the green bands representing the d_{xy} / d_{yz} / d_{xz}. Given those plots, we have $\langle \epsilon_k \rangle$=2.31 and 2.29 eV for undistorted and distorted structures, which lead to ST=1.55

and 1.95 for undistorted and distorted structures. Both of them meet Stoner criterion. In addition, it seems that this structural distortion promotes the Stoner ferromagnetism.

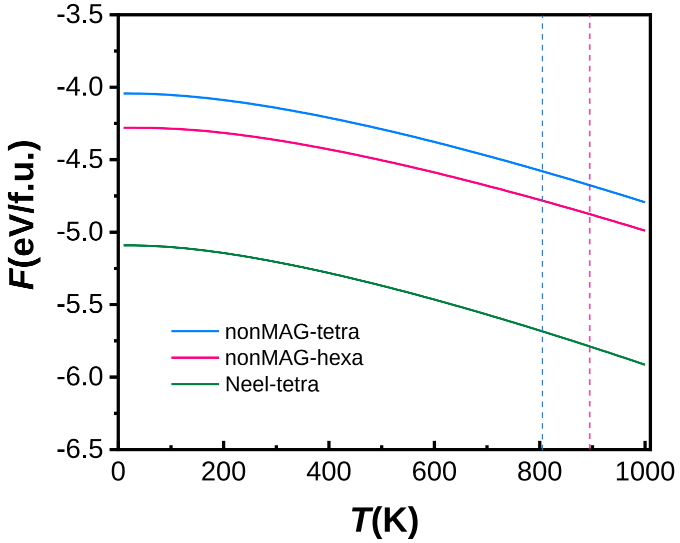

Supplementary Figure 16. Temperature-dependent vibrational free energy. As discussed in the main text, the tetragonal and hexagonal phases are, most likely, paramagnetic (PM) and non-magnetic (NM), respectively. We thus show the calculated free energies of the NM tetragonal and hexagonal phases and the Neel AFM tetragonal phase using blue, red and green lines, respectively. All free energies were estimated by considering vibrational entropy while detailed formulism was documented in literature[2]. Blue and red dashed lines indicate T_{growth} of the tetragonal (T1) and hexagonal (T2) phases, respectively. Here, the PM phase is modeled by an associated Neel AFM phase. According to our calculated results, the NM hexagonal phase is ~ 0.24 eV/f.u. more stable than the NM tetragonal phase, but 0.81 eV/f.u. less stable than Néel AFM tetragonal phase at 0 K. The hexagonal phase seems to have smaller entropy energy in comparison with the tetragonal phase. As a result, there is no crossover of free energies occurred from 0 K up to 1000 K; this indicates the prepared hexagonal phase under a higher Te source temperature deserves another explanation.

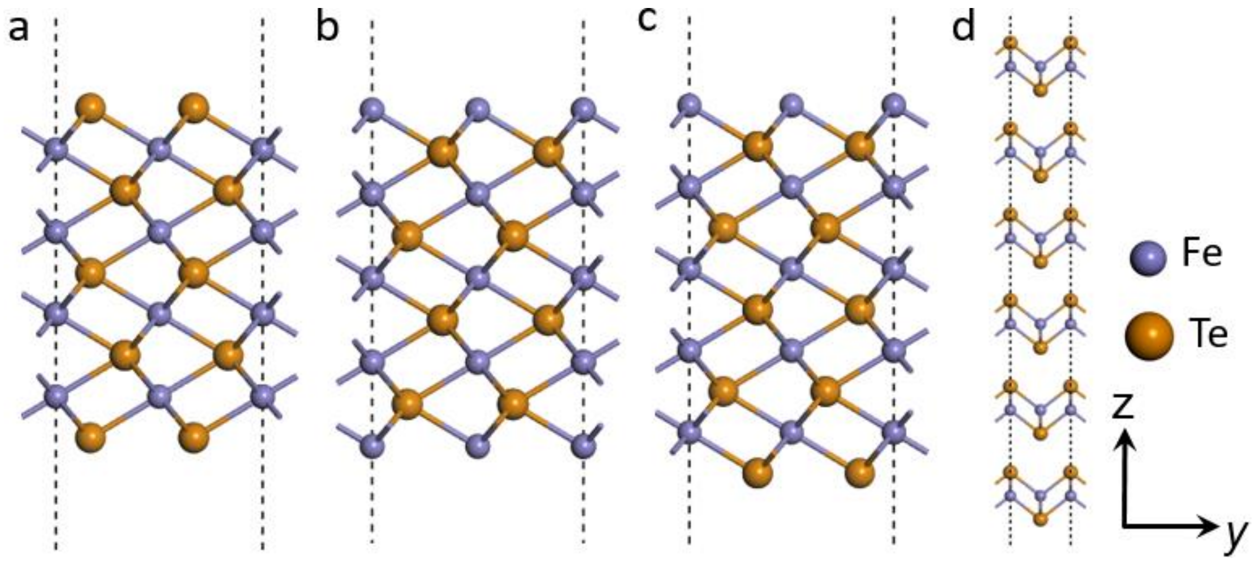

Supplementary Figure 17. Top and side view of the surface configurations. Slab models were used to calculate surface energies of the hexagonal (a-c) and tetragonal phases (d), respectively. Three particular surfaces were considered for the NM hexagonal phase, *i.e.* symmetric Te-terminated (a) and Fe-terminated (b) surfaces and an asymmetric surface (c) with a 1:1 Fe-Te ratio. Here, the surface energy is defined as $(E_{slab} - n \times E_{bulk}) / 2A$ for the asymmetric surface and $(E_{slab} - n_i \times \sum_i \mu_i) / 2A$ for the symmetric surface. Energies E_{slab} and E_{bulk} are the total energies of a surface slab and the associated bulk unit cell, n is the number of formula cells that the surface slab contains, A is the area of the surface, n_i represents the number of the ith element and μ_i denotes the chemical potential of the ith element, representatively. According to our calculation, the surface energies of the NM and Neel AFM Tetragonal phases are 12.8 meV/Å^2 and 10.7 meV/Å^2, respectively. The Fe-terminated and asymmetric hexagonal slabs are rather unstable, with surface energies over 80 meV/Å^2. The Te-terminated hexagonal surface is exceptionally stable that shows a surface energy 12.1 or 1.9 meV/Å^2 for the Fe- or Te-rich limit, respectively, indicating a high probability of obtaining a Fe_xTe_{x+1} slab under a Te-rich growth condition.

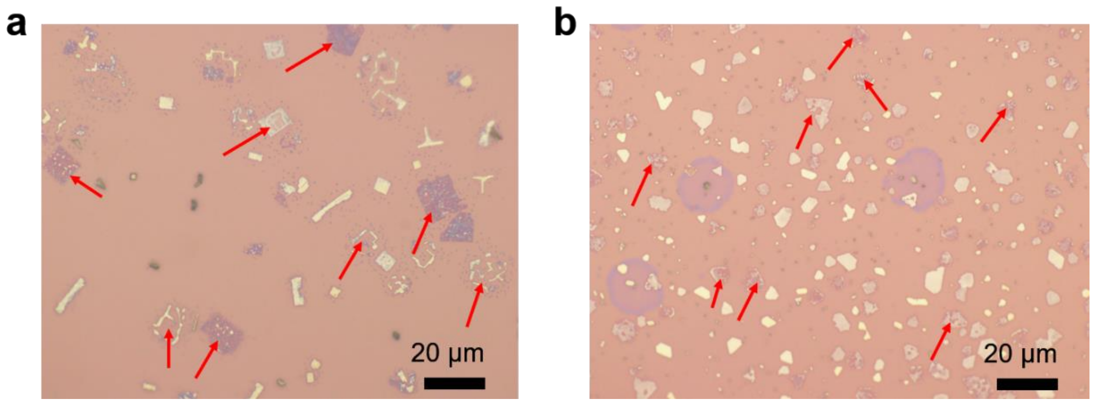

Supplementary Figure 18. Optical images of tetragonal phase FeTe (a) and hexagonal phase FeTe (b) when the furnace was natural cooling.

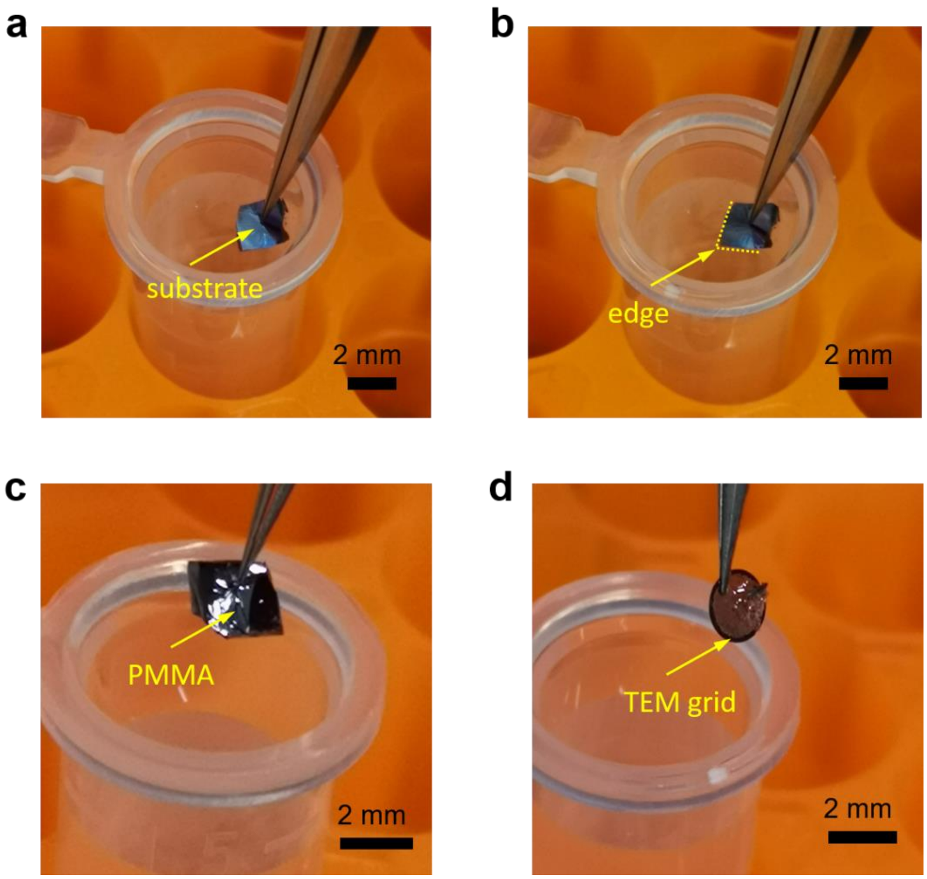

Supplementary Figure 19. a-d The surface-energy-assisted transfer process.

Supplementary Reference

1. Shivayogimath, A., et al. A universal approach for the synthesis of two-dimensional binary compounds. *Nat. Commun.* **10**, 2957 (2019).
2. Duerloo K-AN, Li Y, Reed EJ. Structural phase transitions in two-dimensional Mo- and W-dichalcogenide monolayers. *Nat. Commun.* **5**, 4214 (2014).
